# Tumour-derived TGF-*β*1 modulates myofibroblast differentiation and promotes HGF/SF-dependent invasion of squamous carcinoma cells

**DOI:** 10.1038/sj.bjc.6601611

**Published:** 2004-02-17

**Authors:** M P Lewis, K A Lygoe, M L Nystrom, W P Anderson, P M Speight, J F Marshall, G J Thomas

**Affiliations:** 1 Eastman Dental Institute, University College, London, UK; 2 Department of Tumour Biology, Cancer Research UK, QMW, London, UK

**Keywords:** squamous carcinoma, TGF-*β*1, invasion, scatter factor, hepatocyte growth factor, myofibroblast, stroma

## Abstract

The development of an altered stromal microenvironment is a common feature of many tumours including squamous cell carcinoma (SCC), and there is increasing evidence that these changes in the stroma, which include increased expression of proteases and cytokines, may actually promote tumour progression. A common finding is that stromal fibroblasts become ‘activated’ myofibroblasts, expressing smooth muscle actin and secreting cytokines, proteases and matrix proteins. We show that myofibroblasts are commonly found in the stroma of oral SCC and are often concentrated at the invasive margin of the tumour. Using oral SCC cells and primary oral fibroblasts, we demonstrate that tumour cells directly induce a myofibroblastic phenotype, and that this transdifferentiation is dependent on SCC-derived TGF-*β*1. In turn, myofibroblasts secrete significantly higher levels of hepatocyte growth factor/scatter factor compared with fibroblast controls, and this cytokine promotes SCC invasion through Matrigel, a mixture of basement membrane proteins. This is the first time that this double paracrine mechanism has been demonstrated between squamous carcinoma cells and fibroblasts, and emphasises that cancer invasion can be promoted indirectly by the release of tumour-induced host factors from stroma.

Historically, most studies of neoplastic transformation and progression have focused on the tumour cell. However, in addition to transformed cells, tumours are also composed of host stromal tissue comprising fibroblasts, newly formed blood vessels, extracellular matrix and immune components. Although stroma was initially thought to support tumour development passively, there is increasing evidence to suggest that it actively contributes to malignant progression (Liotta and Kohn, 2001; Pupa *et al*, 2002).

A common finding in many types of solid tumours is that stromal fibroblasts become ‘activated’ and express a number of contractile proteins, particularly *α*-smooth muscle actin (SMA) (Tlsty and Hein, 2001). These cells have been referred to as peritumour fibroblasts, carcinoma-associated fibroblasts and activated stroma, but are now more commonly called myofibroblasts (MF). The process of activation of fibroblasts is associated with increased proliferation, increased deposition of collagen and spliced-variant forms of fibronectin, assembly of vinculin-containing fibronexus adhesion complexes and acquisition of smooth muscle cell characteristics. In fact, it is the expression of SMA that is the hallmark of the myofibroblastic phenotype. The structural changes, such as assembly of fibronexi, and accumulation of cytoskeletal SMA, modulate MF contractility and reduce their migratory potential (Serini and Gabbiani, 1999). Conversely, myofibroblasts upregulate the secretion of numerous growth factors, chemokines and cytokines, as well as extracellular matrix proteins and proteases (Powell *et al*, 1999a, 1999b).

A number of cytokines including PDGF, IL-4, insulin-like growth factor II and TGF-*β*1 may be involved in the transdifferentiation of fibroblasts to myofibroblasts, and these can be derived from a number of different cell types (Powell *et al*, 1999a, 1999b). Among these cytokines, TGF-*β*1 is considered to have a central role in inducing the myofibroblastic phenotype, because it is capable of upregulating fibroblast SMA and collagen both *in vitro* and *in vivo* (Tuxhorn *et al*, 2001). Indeed, high levels of the cytokine are usually associated with MF-containing lesions (Tuan and Nichter, 1998). In many types of cancers, TGF-*β*1 is overexpressed by carcinoma cells (Ronnov-Jessen *et al*, 1996; Rowley, 1998), and it has been proposed previously that the expression of this cytokine by breast and prostate carcinoma cells induces reactive stroma (Ronnov-Jessen *et al*, 1996; Rowley, 1998; Webber *et al*, 1999). TGF-*β*1 has many effects: In addition to inhibiting epithelial cell proliferation, it also promotes the secretion of matrix proteins and proteases. Its powerful antiproliferative effect has led to it being thought of as a tumour suppressor in carcinomas. However, it is now apparent that TGF-*β*1 may be pro-oncogenic, driving malignant progression, invasion and metastasis (Wakefield and Roberts, 2002). This is partly explained by observations of carcinomas, including oral SCC, which become refractory to the antiproliferative effect of TGF-*β*1. However, another mechanism by which TGF-*β*1 could promote tumour development is by inducing the transdifferentiation of stromal fibroblasts, producing an activated, myofibroblast-rich stromal microenvironment. For example, it has been shown that TGF-*β*1 produced by breast cancer cells activates normal breast stromal fibroblasts and promotes them to produce urokinase-type plasminogen activator, a serine protease important in cancer cell invasion and metastasis (Sieuwerts *et al*, 1998). Such changes have a potential role in tumorigenesis since, if tumour stroma becomes activated and immobilised in the vicinity of tumour cells, paracrine interactions may be established between the separate cellular compartments, some of which could encourage tumour development.

To date, there has been little work investigating potential interactions between squamous carcinomas (SCC) and the surrounding stroma. Maas-Szabowski *et al* (2001) showed that IL-1 produced by epidermal keratinocytes induced the expression of keratinocyte growth factor by dermal fibroblasts, which in turn stimulated keratinocyte proliferation. It has also been suggested that PDGF-activated stromal cells may maintain elevated keratinocyte proliferation via a paracrine mechanism (Skobe and Fusenig, 1998). Ramos *et al* (1997) demonstrated that peritumour fibroblast-conditioned medium promoted SCC migration on tenascin, and that this effect could be partially inhibited by blocking EGF, TGF-*β*1 or hepatocyte growth factor/scatter factor (HGF/SF). In addition, paracrine interactions have been demonstrated between squamous carcinoma cells and other cell types found in stroma. Liss *et al* (2001) found tumour-derived TGF-*β*1 and monocyte chemotactic protein-1 attracted and activated monocytes. They suggested that macrophages secreted TNF-alpha and IL-1, which in turn stimulated tumour cells to produce IL-8 and VEGF, the latter cytokine then inducing angiogenesis.

The aim of the study was to investigate the role of squamous carcinoma cells in myofibroblast transdifferentiation, to determine the effect of such cells on SCC invasion and to elucidate the possible mechanisms involved in these processes.

We show that myofibroblasts are commonly found within the stroma of squamous carcinoma *in vivo*, particularly at the invasive front. We demonstrate that squamous carcinoma cells may directly induce a myofibroblast phenotype in primary fibroblasts through the secretion of TGF-*β*1. Furthermore, such transdifferentiated myofibroblasts significantly upregulate the secretion of hepatocyte growth factor (HGF/SF), which promotes SCC invasion through basement membrane proteins. These *in vitro* data are consistent with the possibility that a similar double paracrine effect may also exist *in vivo*.

## MATERIALS AND METHODS

### Antibodies and reagents

Six monoclonal antibodies (mAbs) (all of mouse origin) were used in this study. Antibodies were purchased against human TGF-*β*1, HGF/SF (R&D Systems, Abingdon, UK), c-met and phosphorylated c-met (Upstate Ltd, Milton Keynes, UK) and SMA (Sigma, Dorset, UK; DAKO, High Wycombe, UK). W632 (anti-MHC class I) was a kind gift from W Bodmer (IMM, Oxford). FITC-conjugated rabbit anti-mouse immunoglobulin was purchased from DAKO (High Wycombe, UK). Recombinant human TGF-*β*1 was purchased from R&D Systems, Abingdon, UK. Matrigel was obtained from Becton Dickinson (Oxford, UK).

### Immunohistochemistry

Sections (3 *μ*m) were dewaxed, brought to absolute alcohol and endogenous peroxidase neutralised with 0.5% methanolic hydrogen peroxide for 10 min. Sections were washed in water, followed by 0.05% Tween 20 in TBS pH 7.4 (TBS/Tween). Primary anti-SMA antibody was applied for 60 min at a dilution of 1 : 150 (Dako, High Wycombe, UK). Sections were again washed in TBS/Tween and secondary antibody applied for 30 min (Dako K5001 ChemMate HRP/DAB kit). Sections were washed in TBS/Tween and peroxidase-labelled streptavidin was applied for 30 min (Dako K5001). The peroxidase was visualised using DAB (Dako K5001) for 7 min and counterstained in Mayer's haematoxylin. In all, 15 archival oral SCCs and 10 benign polyps showing fibroepithelial hyperplasia were chosen at random, stained for SMA and scored by two pathologists independently (PMS and GJT), according to the Quickscore method (Lee *et al*, 2002). Briefly, the staining intensity was scored out of 3 (1=weak, 2=moderate, 3=strong), and the proportion of the stroma in or adjacent to the tumour staining positively was scored out of 4 (1=<25%, 2=25–50%, 3=51–75%, 4=76–100%). The score for intensity was added to the score for proportion to give a score in the range of 0–7 and grouped as –(score=0), +(score=1–3), ++(score=4–5) or +++(score=6–7). Pathologists agreed completely in 11 of the 15 cases of SCC. The remaining four cases were reanalysed and a consensus score agreed. Staining of benign polyps for SMA-positive myofibroblasts was uniformly negative.

### Cell culture

Human primary oral fibroblasts (OF) had been established previously in the laboratory from redundant human tissue obtained during routine periodontal surgery at the Eastman Dental Hospital. Oral fibroblasts were maintained in fibroblast growth medium. This consisted of D-MEM (Life Technologies, Gibco BRL, Paisley, UK) supplemented with 10% foetal calf serum (FCS; PAA Laboratories, Yeovil, UK) plus penicillin (100 U ml^−1^) and streptomycin (100 *μ*g ml^−1^) (Life Technologies). Cells were maintained in a humidified atmosphere of 5% CO_2_ at 37°C and routinely passaged using tryspin–EDTA (Life Technologies). A panel of three oral SCC cell lines were used. We generated the invasive VB6 cell line previously by transfection and retroviral infection of integrin subunits to express high levels of the integrin *α*v*β*6 (Thomas *et al*, 2001b). CA1 and 5PT were kind gifts from Professor IC Mackenzie (Cardiff dental School, UK). Cells were grown in standard keratinocyte growth medium (KGM) as described (Sugiyama *et al*, 1993). Keratinocyte growth medium comprised *α*-MEM containing 10% FCS (Globepharm, Surrey) supplemented with 1.8 × 10^−4^ M adenine, 5 *μ*g ml^−1^ insulin, 0.5 *μ*g ml^−1^ hydrocortisone and 10 ng ml^−1^ epidermal growth factor (Sigma).

### Preparation and use of medium conditioned by SCCs (SCCM)

Squamous carcinoma cells were grown to 70% confluence in KGM in 80 cm^2^ culture flasks, washed twice with phosphate-buffered saline (PBS; Life Technologies) and incubated for 72 h with 10 ml of *α*-MEM. The SCCM from each cell line was collected, clarified by centrifugation and the cells were detached with trypsin/EDTA and counted. A total of 1.5 × 10^3^ OF cm^−2^ were plated in fibroblast growth medium in 80 cm^2^ culture flasks or on glass coverslips for 3 days, then washed twice with PBS and incubated for 72 h with *α*-MEM, SCCM (at equal volumes keratinocyte cell number^−1^) or *α*-MEM containing TGF-*β*1 (R&D Systems, Abingdon, UK; 10 ng ml^−1^). For blocking studies, anti-TGF-*β*1 antibody (R&D Systems; 1 *μ*g ml^−1^) or a control antibody (W632; anti-MHC type 1; 1 *μ*g ml^−1^) was added to the SCCM for 30 min prior to incubation with the fibroblasts. All experiments were performed in triplicate and the experiments were repeated four times.

### Preparation and use of medium conditioned by fibroblasts and myofibroblasts

A total of 1.5 × 10^3^ fibroblasts cm^−2^ were plated in fibroblast growth medium in 80 cm^2^ culture flasks for 3 days and then washed twice with PBS. To induce a myofibroblast, phenotype cells were incubated for 72 h with *α*-MEM containing recombinant TGF-*β*1 (R&D Systems, Oxford, UK), which was acid-activated prior to use (4 mM HCl/0.1% BSA). TGF-*β*1 was titrated at concentrations ranging from 0.5 to 10 ng ml^−1^, with maximum SMA induction observed at 10 ng ml^−1^. This concentration was then used routinely in all experiments. Control cells were cultured in *α*-MEM alone. Cells were also incubated for 72 h with SCCM (at equal volumes/keratinocyte cell number). The cells were washed twice with PBS and cultured for a further 72 h in *α*-MEM. The control fibroblast- (FCM) or myofibroblast-conditioned medium (MCM) was collected, clarified by centrifugation and the cells were detached and counted. The volumes of FCM and MCM were corrected for cell number, adjusted to a total volume of 500 *μ*l and used in the lower chamber of a Transwell invasion assay as a chemoattractant, or assayed by ELISA for HGF/SF.

### Immunoflourescence microscopy

Primary fibroblasts grown on glass coverslips were treated with SCCM and *α*-MEM (±TGF-*β*1) for 72 h. For blocking studies, anti-TGF-*β*1 antibody (R&D Systems; 1 *μ*g ml^−1^) or a control antibody (W632; anti-MHC type 1; 1 *μ*g ml^−1^) was added to the SCCM for 30 min prior to incubation with the primary fibroblasts. The cells were prefixed in 2% paraformaldehyde (BDH), rinsed in PBS, fixed with methanol (BDH) for 10 min at −20°C, permeabilised in 0.25% Triton (Sigma) in PBS and labelled by indirect immunostaining. Primary anti-SMA antibody (Sigma, clone IA4) was used at a concentration of 1 : 1000, while the secondary antibody was FITC-conjugated rabbit anti-mouse immunoglobulin (Dako, High Wycombe, UK; 1 : 500). Nuclei were visualised using DAPI (Sigma). Images were captured using a Cohu CCD camera attached to a Leica DM IRB microscope (Leica Microsystems (UK) Ltd, Milton Keynes, UK).

### ELISA

ELISA kits for TGF-*β*1 and HGF/SF were purchased from R&D Systems (Oxford, UK). The assay is based on a two site ELISA ‘sandwich’ format. Cell supernatants were prepared as for conditioned medium and TGF-*β*1 activated by adding 0.1 ml of 1 M HCL for 10 min. This was neutralised with 100 *μ*l of 1.2 M NaOH/0.5 M HEPES. Sample (200 *μ*l) was added to each well and TGF-*β*1 or HGF/SF detected by a peroxidase-labelled FAb′ antibody directed to either cytokine. The reaction was stopped by the addition of an acid solution and the resultant colour change was read at 450 nm on a spectrophotometer. The concentration was determined by interpolation from a standard curve using known concentrations of TGF-*β*1 or HGF/SF standards as supplied.

### Immunoblotting

Cells of equal confluence were lysed with SDS lysis buffer containing protease inhibitors (1% SDS, 10 mM Tris, pH 7.4, leupeptin 100 *μ*g ml^−1^, phenylmethylsulphonyl fluoride 100 *μ*g ml^−1^, aprotinin 100 *μ*g ml^−1^) and the protein was estimated using the BCA protein assay reagent (Pierce Warriner). Samples containing equal protein were boiled in reducing buffer (0.5 M Tris-HCl pH 6.8, 10% SDS, 10% glycerol, 0.4% bromophenol blue, 10% *β*-mercaptoethanol) and electrophoresed in 10% SDS–PAGE gel. Protein was electrotransferred onto nitrocellulose membranes (Hybond-C, Amersham, UK) in transfer buffer (20 mM glycine, 25 mM Tris, 0.6 mM SDS, 10% methanol) for 12 h at 26 mV. To prevent nonspecific binding, blots were blocked for 1 h at room temperature in 5% skimmed milk powder (Marvel®, Cadbury, UK) in PBS 0.1% Tween. Anti-SMA antibody (Sigma; clone 1A4; 1 : 1000 dilution), anti-c-met antibody (Upstate Ltd, UK; 1 : 750 dilution) and antiphosphorylated c-met (Upstate Ltd, UK; 1 : 500 dilution) were used for immunoblotting. Horseradish peroxidase-conjugated anti-mouse was used as secondary antibody at a 1 : 2000 dilution. Blots were developed with the ECL Western blotting detection kit system (Amersham, UK). Blots were also probed for *β*-actin as an additional loading control. These experiments were repeated a minimum of three times.

### Invasion assays

Cell invasion assays were performed using Matrigel-coated polycarbonate filters (8 *μ*m pore size, Transwell®, Beckton Dickinson) as described previously (Thomas *et al*, 2001a). Matrigel (70 *μ*l; 1 : 2 dilution in *α*-MEM) was added to the upper membrane and allowed to gel for 1 h at 37°C. Fibroblast conditioned medium or MCM was corrected for cell number, adjusted to a final volume of 500 *μ*l with *α*-MEM and used as a chemoattractant in the lower chamber of the Transwell. For blocking experiments, the conditioned media were incubated with anti-HGF/SF antibody (R&D Systems; 10 *μ*g ml^−1^) for 30 min at 4°C prior to placing in the assay. An irrelevant antibody (W632; anti MHC-type I; 10 *μ*g *μ*l^−1^) was used as a control. Squamous carcinoma cells were plated in the upper chamber of quadruplicate wells at a density of 5 × 10^4^ in 200 *μ*l of *α*-MEM and incubated at 37°C for 72 h. The cells in the lower chamber (including those attached to the undersurface of the membrane) were then trypsinised and counted on a Casy 1 counter (Sharfe System GmbH, Germany). Experiments were repeated four times in quadruplicate.

### Statistical analysis

Data are expressed as the mean±s.d. of a given number of observations. Where appropriate, one-way analysis of variance (ANOVA) was used to compare multiple groups. For comparisons between groups, Fisher's PLSD (set at 5% significance) was used. A *P*-value of <0.05 was considered to be significant.

## RESULTS

### Myofibroblastic differentiation is commonly seen in the stroma of SCCs *in vivo*, particularly at the invasive front of the tumour

Of the 15 oral SCCs examined, 11 (73%) contained a significant proportion of strongly SMA-positive stromal cells, indicating myofibroblastic differentiation (Table 1

**Table 1:** SMA staining intensity was assessed semiquantitatively using the Quick score (range 1–7) (Lee *et al*, 2002) by two pathologists independently (PMS and GJT)

	**Site**	**Differentiation**	**Primary, tumour, size**^a^	**Stage**	**SMA expression**
1	FOM	Mod	T4	IV	+++
2	Tongue	Mod	T2	II	+++
3	FOM	Mod	T4	IV	+++
4	Tongue	Well	T3	III	+
5	FOM	Mod	T4	IV	++
6	Retromolar	Poor	T4	IV	−
7	Alveolar mucosa	Mod	T4	IV	++
8	Tongue	Poor	T4	IV	++
9	FOM	Mod	T4	IV	++
10	FOM	Mod	T4	IV	++
11	Tonsillar fossa	Poor	T4	IV	+++
12	Tongue	Mod	T3	IV	+++
13	Tongue	Mod	T1	III	+
14	FOM	Mod	T4	IV	++
15	Tongue	Mod	T1	IV	+

**Figure 1:**
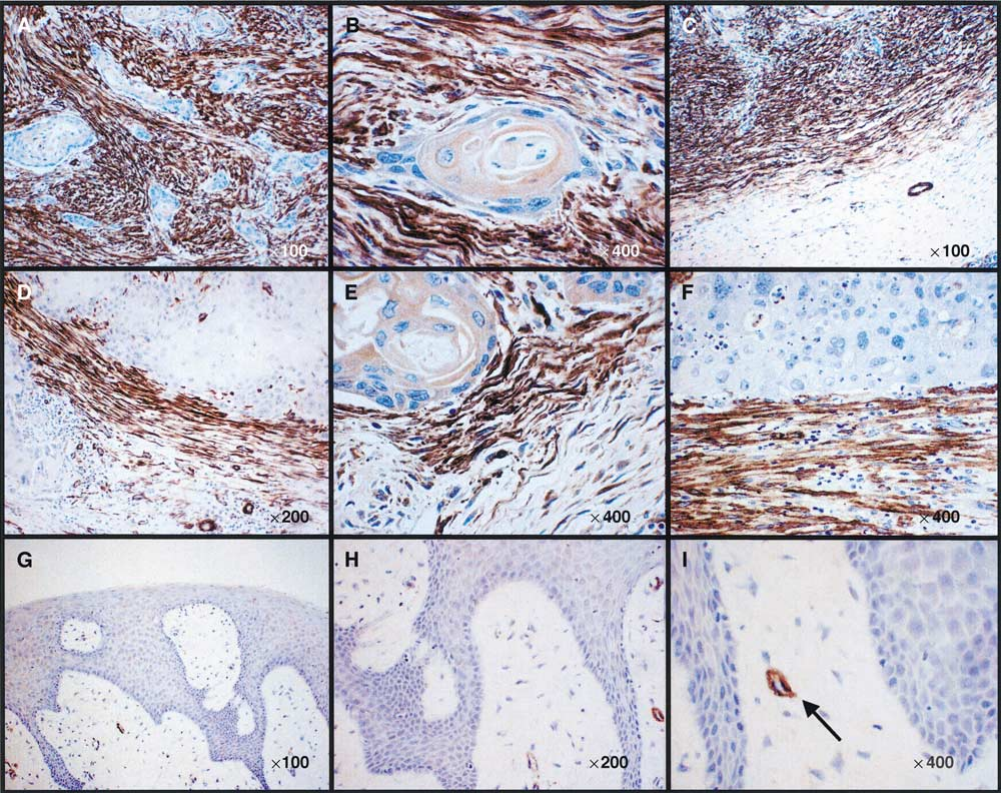
Stroma of oral SCC contains prominent myofibroblasts *in vivo*. Immunohistochemistry showing SMA expression by myofibroblasts in oral SCC and lack of expression in benign fibroepithelial hyperplasia. (**A**) Islands of SCC scattered throughout a myofibroblastic stroma with prominent SMA expression (magnification= × 100). (**B**) A single island of SCC surrounded by SMA-positive myofibroblasts (magnification= × 400). (**C**) Smooth muscle actin expression is concentrated at the tumour margin. Strong SMA expression by myofibroblasts is only detected in the near vicinity of the tumour. Consequently, the margin of the carcinoma appears sharply defined where it abuts ‘normal’ fibroblastic tissue (magnification= × 100). (**D**–**F**) Strong induction of SMA expression immediately adjacent to islands of SCC (magnification= × 200, × 400, × 400, respectively). This was usually seen adjacent to the invasive margin at the tumour periphery. (**G–I**) Lack of SMA expression in fibroblasts of benign fibroepithelial hyperplasia (magnification= × 100, × 200, × 400, respectively). The arrow in (**I**) indicates positive SMA staining of smooth muscle in the wall of a blood vessel, which serves as an internal positive control.

The SMA staining intensity was scored out of three (1=weak, 2=moderate, 3=strong) and the positive proportion of the stroma in or adjacent to the tumour was scored out of four (1=<25%, 2=25–50%, 3=51–75%, 4=76–100%). The score for intensity was added to the score for proportion to give a score in the range of 0–7 and grouped as −(score=0),+(score=1–3), ++(score=4–5) or +++(score=6–7). Of the 15 tumours examined, 11 (73%) contained a significant proportion of strongly SMA-positive stromal cells, indicating myofibroblastic differentiation.

a Tumour size T1=<20 mm; T2=20–40 mm; T3=>40 mm, T4=tumour with extension to involve adjacent structures. SMA=*α*-smooth muscle actin. FOM=floor of mouth.

). Four tumours contained focal areas of strong stromal SMA expression. No tumour was completely SMA negative, and myofibroblastic stromal differentiation was seen in all tumour grades and stages. Although variation in myofibroblast distribution was seen between different tumours, this did not correlate with tumour grade or architecture. Figure 1

shows the prominence of myofibroblasts in the tumour stroma. Such cells were usually concentrated at the invasive margin of the tumour, directly abutting malignant epithelial cells (Figure 1C). Stromal SMA expression often demarcated the margin of the tumour (Figure 1C) and was only observed in close proximity to the tumour mass (Figure 1D), even in tumours containing a diffuse inflammatory infiltrate. This suggests that in oral SCC myofibroblasts may be induced primarily by tumour cells. In addition, 10 benign mucosal polyps were stained for SMA expression to determine whether hyperplastic (but non-malignant) squamous epithelium could also induce SMA induction in adjacent fibroblasts. Although positive staining of blood vessel smooth muscle was observed (Figure 1I arrow), no polyp contained SMA-positive myofibroblasts in the connective tissue (Figure 1(G, H and I).

### Squamous carcinoma cells induce myofibroblast differentiation through secretion of TGF-*β*1

**Figure 2:**
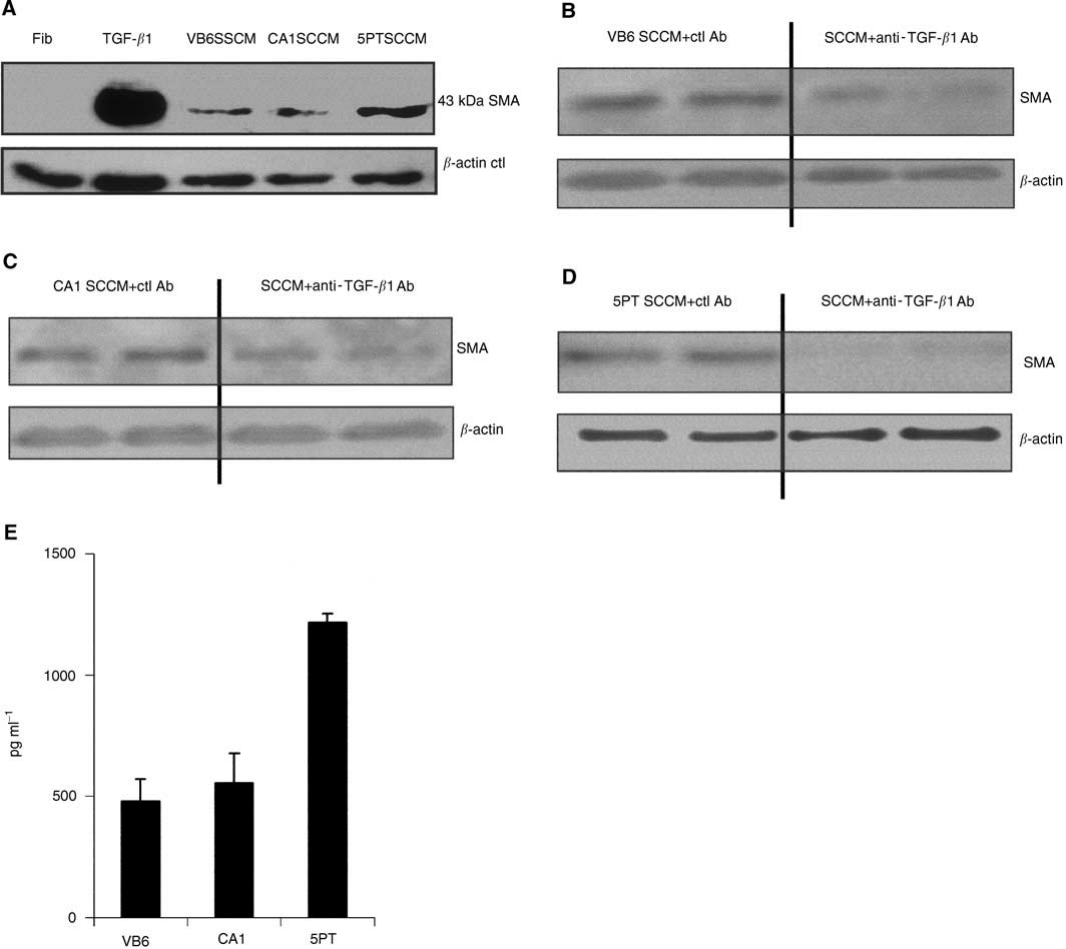
Squamous carcinoma-conditioned medium (SCCM) induces fibroblast SMA expression that is TGF-*β*1 dependent. (**A**) Western blot showing SMA expression by fibroblasts (Fib) compared with myofibroblasts, which had been generated by culture with exogenous recombinant TGF-*β*1 or SCCM from the SCC cell lines, *β*-actin was used as a loading control. (**B–D**) Western blots showing that the induction of SMA expression by SCCM from VB6 (**B**), CA1 (**C**) and 5PT (**D**) cells could be inhibited significantly by a TGF-*β*1 blocking antibody. An irrelevant antibody (W632; anti-MHC type I) was used as a control. This confirmed that generation of the myofibroblastic phenotype was TGF-*β*1 specific. The figures show representative experiments. *β*-actin was used as a loading control. (**E**) ELISA confirming that the SCC cell lines secrete TGF-*β*1. Squamous cell carcinoma cell lines VB6, CA1 and 5PT were cultured in *α*-MEM for 72 h. The conditioned medium was adjusted for cell number, acid activated and analysed by ELISA for total TGF-*β*1. 5PT cells secreted significantly higher levels of TGF-*β*1 compared with VB6 and CA1 cells. The figure shows representative experiments performed in triplicate. Error bars represent s.d.

Human gingival fibroblasts expressed low levels of SMA in culture (Figure 2A

, 3A

**Figure 3:**
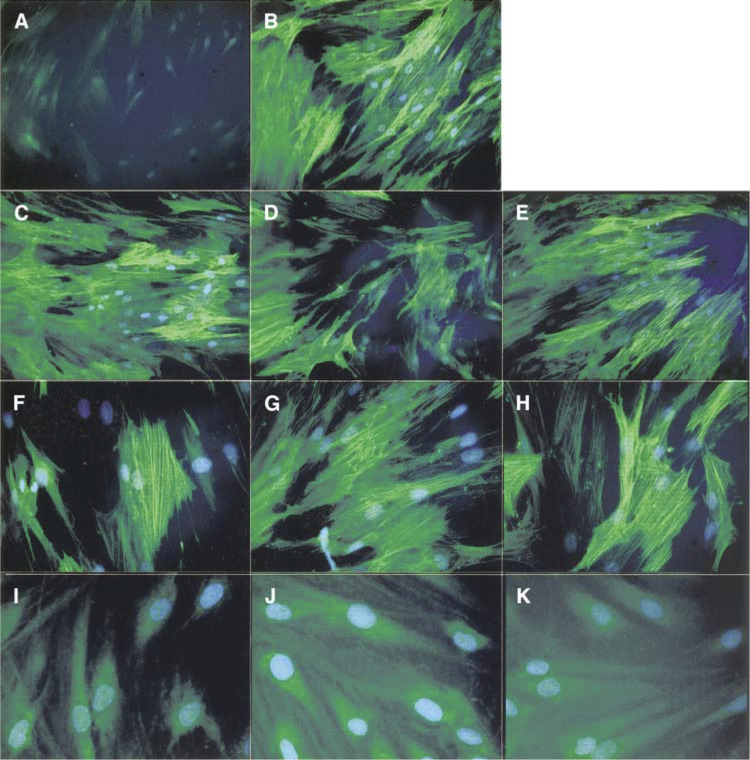
Squamous carcinoma conditioned medium induces fibroblast SMA expression that is TGF-*β*1 dependent. Fibroblasts grown on glass coverslips were treated for 72 h with *α*-MEM, *α*-MEM containing TGF-*β*1 (10 ng ml^−1^) or SCCM from each cell line. For blocking studies, anti-TGF-*β*1 antibody (1 *μ*g ml^−1^) was added to the SCCM for 30 min prior to treating the fibroblasts. Smooth muscle actin (green staining) was detected using mouse mAb IA4 (Sigma) with an FITC-conjugated rabbit anti-mouse secondary antibody (Dako). Nuclei (blue staining) were visualised using DAPI (Sigma). Images were captured using a Leica DC200 digital camera attached to a Leica DM IRB microscope (Leica Microsystems (UK) Ltd, Milton Keynes, UK). Magnification= × 20 (**A–E**), × 40 (**F–H**), × 60 (**I–K**). The figure shows occasional weak diffuse cytoplasmic SMA staining in control fibroblasts treated with *α*-MEM only (**A**). SMA expression is strongly upregulated in fibroblasts treated with recombinant TGF-*β*1 (10 ng ml^−1^; **B**) or SCCM from each cell line (VB6=**C**, **F**; CA1=**D**, **G**; 5PT=**E**, **H**). In each instance, SMA is organised into stress fibres. The addition of inhibitory TGF-*β*1 antibody to SCCM prevented SMA upregulation (VB6=**I**, CA1=**J**, 5PT=**K**), confirming that the induction of the myofibroblast phenotype was TGF-*β*1 specific.

). Immunostaining showed occasional cells with weak, diffuse cytoplasmic expression of the protein (Figure 3A). Treatment of primary fibroblasts with exogenous-activated TGF-*β*1 (at concentrations ranging from 0.5 to 10 ng ml^−1^) produced a significant increase in SMA expression (data not shown). Maximum SMA expression was observed at a concentration of 10 ng ml^−1^ (Figures 2A and 3B). Figure 3B demonstrates increased intensity of SMA staining and shows that the protein is now associated with cytoplasmic stress fibres. SMA upregulation was also observed when primary fibroblasts were cultured in conditioned medium from VB6, CA1 and 5PT cell lines squamous carcinoma cell lines (SCCM) (Figures 2A and 3C–H).

ELISA on SCCM confirmed that the SCC cell lines produced TGF-*β*1 (Figure 2E), the highest levels secreted by 5PT cells. In order to determine the proportion of activated TGF-*β*1 in the SCC supernatants, we repeated the ELISA comparing acid-activated conditioned medium (in which all TGF-*β*1 is in active form) with untreated samples. In conditioned media from VB6, CA1 and 5PT cells, we found that the proportion of activated TGF-*β*1 relative to total TGF-*β*1 was 87, 81 and 59%, respectively. In addition, however, fibroblasts probably activate TGF-*β*1, and thus the initial amount of activated cytokine in the SCC medium, before it is placed onto the fibroblasts, may not be relevant to its eventual biological effect.

To demonstrate that the generation of a myofibroblastic phenotype was TGF-*β*1-dependent, we carried out blocking studies using a TGF-*β*1 inhibitory antibody that blocks the biological activity of activated TGF-*β*1. Figures 2B–D and 3I–K demonstrate that when TGF-*β*1 inhibitory antibody was added to SCCM from VB6, CA1 and 5PT cells prior to fibroblast treatment, the induction of SMA expression was reduced significantly (as determined by densitometric scanning; by 65%, *P*=0.0297; 75%, *P*=0.0028; 82%, *P*=<0.0005, respectively). These data were confirmed by immunofluorescence, which showed only a weak diffuse cytoplasmic staining for SMA with no stress fibre formation when TGF-*β*1 was inhibited (Figure 3I–K).

### MCM promotes invasion of SCC cells

**Figure 4:**
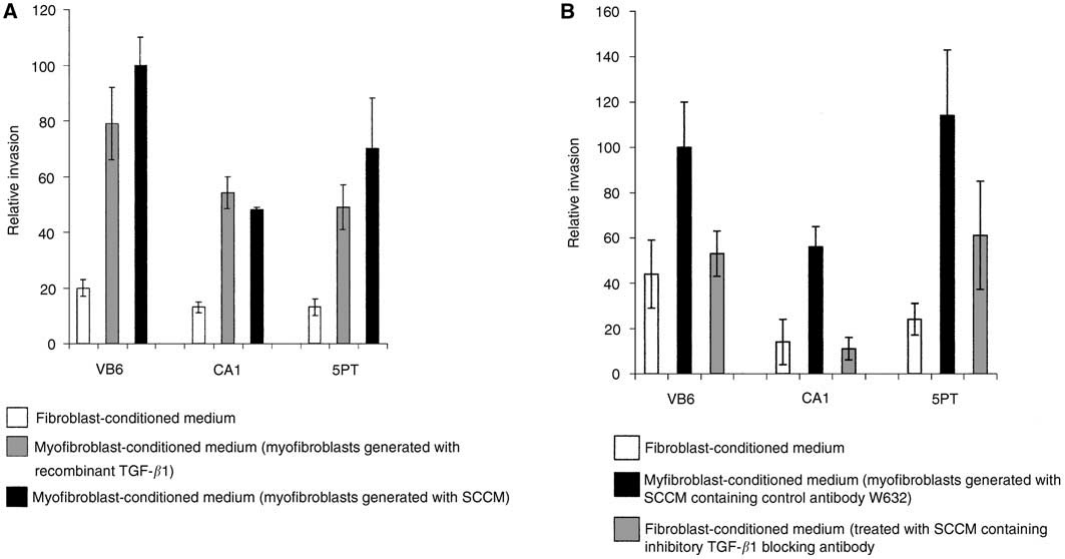
Myofibroblast conditioned medium (MCM) promotes invasion of SCC cells. Cell invasion assays were performed over 72 h using matrigel-coated polycarbonate filters. Conditioned medium from myofibroblasts that had been generated with recombinant TGF-*β*1 (grey-filled histogram) or SCCM (black-filled histogram) was used as a chemoattractant in the lower chamber of the Transwell. This was compared with FCM white-filled histogram). Following incubation, the cells in the lower chamber (including those attached to the undersurface of the membrane) were trypsinised and counted on a Casy 1 counter (Sharfe System GmbH, Germany). The figure shows a representative experiment performed in quadruplicate. Error bars represent s.d. (**A**) Myofibroblast-conditioned medium significantly promoted invasion of VB6, CA1 and 5PT cell lines. In comparison, little invasion was seen when FCM was used. Results are expressed relative to VB6 invasion using SCCM-generated MCM (=100). (**B**) Fibroblasts treated with SCCM containing an inhibitory TGF-*β*1 antibody did not show myofibroblast transdifferentiation. Conditioned medium from such cells (grey-filled histogram) did not promote invasion compared with MCM (black-filled histogram), where the myofibroblasts were generated with SCCM containing a control antibody, W632. Results are expressed relative to VB6 invasion using SCCM-generated myofibroblast conditioned medium (=100).

To determine whether myofibroblasts secrete factors, which stimulate the invasion of squamous carcinoma cell lines, we carried out transwell assays through Matrigel. Myofibroblasts were generated using either TGF-*β*1 or SCCM from each cell line, and then cultured for 72 h in *α*-MEM. The MCM was used as a chemoattractant in the lower chamber of the Transwell and SCC cells were allowed to invade towards this stimulus for 72 h before being counted. Primary FCM was used for comparison. We demonstrate that MCM significantly promoted invasion of VB6, CA1 and 5PT cells compared with FCM (Figure 4A

; *P*=<0.0001, <0.0001, 0.0005, respectively). Myofibroblasts-conditioned medium from myofibroblasts generated using exogenous recombinant TGF-*β*1 (10 ng ml^−1^) produced a similar level of invasion as MCM from myofibroblasts generated by SCCM from each of the cell lines (Figure 4A). If TGF-*β*1 was inactivated using a blocking antibody added to SCCM, no transdifferentiation of myofibroblasts was seen (Figure 3I–K), and conditioned medium from such cells (which remained fibroblasts) no longer promoted invasion (Figure 4B). To ensure that altered cell invasion was not simply due to increased cell proliferation, growth assays were performed in which SCC cells were grown in MCM or FCM for 72 h. The cell proliferation was low (due to the absence of serum in FCM and MCM) and no differences in growth rate were observed.

### Myofibroblasts upregulate secretion of HGF/SF

**Figure 5:**
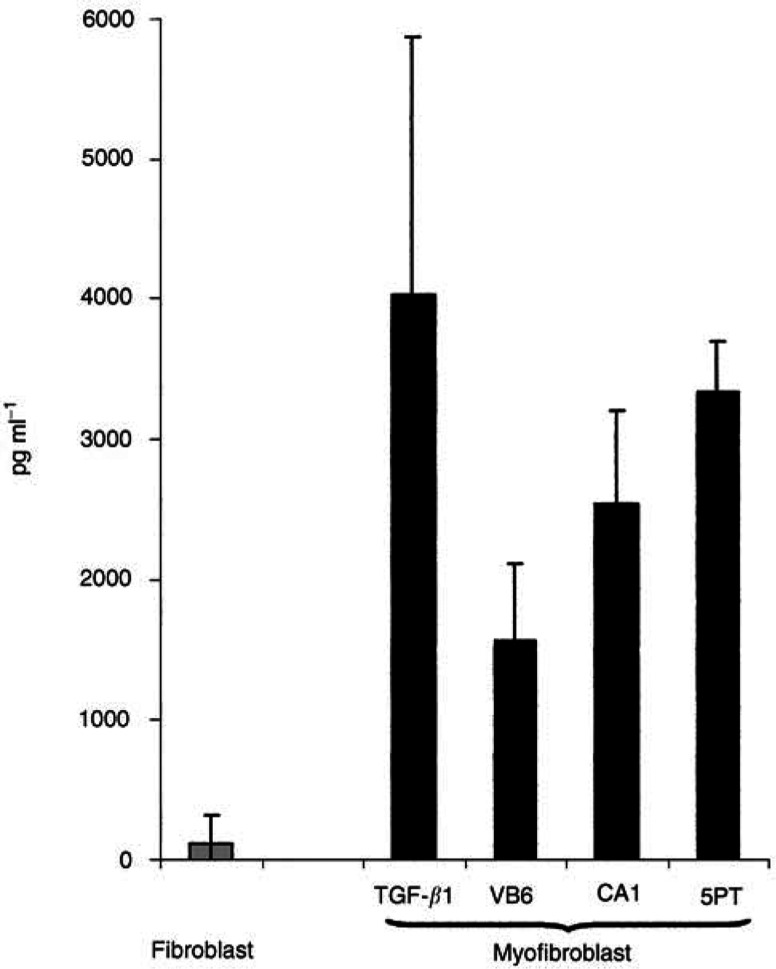
Myofibroblasts upregulate secretion of hepatocyte growth factor (HGF/SF). Myofibroblasts were generated using either exogenous TGF-*β*1 or SCCM from each SCC cell line. Untreated fibroblasts were compared as a control. Supernatants were collected from the cells after 72 h, corrected for cell number and analysed by ELISA for HGF/SF. ELISA demonstrating that MCM contains significantly higher levels of HGF/SF compared with FCM. Myofibroblasts generated using conditioned medium from all three SCC cell lines consistently showed a significant upregulation of HGF/SF secretion when compared with fibroblast controls. The figure shows a representative experiment performed in triplicate. Error bars represent s.d.

Previously, several studies have demonstrated that myofibroblasts may secrete HGF/SF (Bradbury, 1998; Goke *et al*, 1998). This cytokine acts to promote epithelial cell growth and migration and has been shown to stimulate invasion in prostate carcinoma cells (Nishimura *et al*, 1999). To determine whether induction of a myofibroblastic phenotype was associated with increased production of HGF/SF, we examined conditioned medium by ELISA. Myofibroblasts were generated using either exogenous TGF-*β*1 or SCCM from each SCC cell line. Untreated primary fibroblasts were used as a control. Figure 5

demonstrates that MCM contains significantly higher levels of HGF/SF compared with FCM (up to 35-fold higher). Myofibroblasts that had been generated using conditioned medium from all three SCC cell lines consistently showed a significant upregulation of HGF/SF secretion when compared with primary fibroblast controls (VB6, *P*=0.0063; CA1, *P*=0.0003; 5PT, *P*=<0.0001). Secreted HGF/SF levels were generally higher in myofibroblasts, which had been generated with conditioned medium from the 5PT cell line. This was probably due to the higher levels of TGF-*β*1 produced by this line (Figure 2E).

### Inactivation of hepatocyte growth factor in MCM inhibits invasion of squamous carcinoma cells

**Figure 6:**
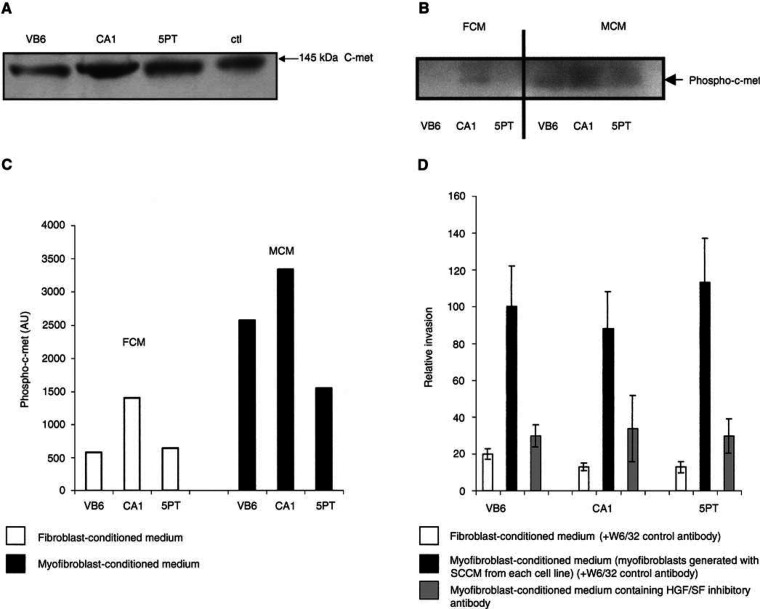
(**A**) *SCC cell lines express c-met receptor*: Western blot showing expression of c-met protein by VB6, CA1 and 5PT cell lines. Control is A431 cell lysate (Upstate, Milton Keynes, UK). (**B**) *Myofibroblast-conditioned medium induces increased phosphorylation of c-met receptor compared with FCM*. Western blot demonstrating increased phosphorylation of c-met in VB6, CA1 and 5PT cells when incubated with MCM compared with FCM. Cells were serum starved for 48 h, incubated with MCM or FCM for 30 min and lysed. Blots were probed with anti-phospho c-met antibody (Upstate Ltd, Milton Keynes, UK), which recognises specifically the phosphorylated form of the receptor. (**C**) Densitometric scan of Western blot confirming increased phosphorylation of c-met in SCC cells treated with MCM. (**D**) *Inactivation of HDF/SF in MCM inhibits invasion of squamous carcinoma cells*. Cell invasion assays were performed over 72 h using matrigel coated polycarbonate filters. Conditioned medium from myofibroblasts, which had been generated with SCCM were treated with a control antibody (W632; black-filled histogram) or an HGF/SF-inhibitory antibody (grey-filled histogram) and used as a chemoattractant in the lower chamber of the Transwell. Fibroblast-conditioned medium (white-filled histogram) was also used in comparison. Following incubation, the cells in the lower chamber (including those attached to the undersurface of the membrane) were trypsinised and counted on a Casy 1 counter (Sharfe System GmbH, Germany). The figure shows a representative experiment performed in quadruplicate. Results are expressed relative to VB6 invasion using SCCM-generated myofibroblast conditioned medium (=100). Error bars represent s.d. The inactivation of HGF/SF in MCM significantly reduced invasion of VB6, CA1 and 5PT cells. Following HGF/SF inhibition, the level of invasion was similar to that produced by FCM, suggesting that the invasion-promoting effect of MCM was modulated through HGF/SF.

To determine whether HGF/SF promoted invasion in the Transwell assays, we carried out blocking experiments using inhibitory antibodies directed against HGF/SF, which were added to the MCM. Figure 6A

confirms that the three SCC cell lines expressed the HGF/SF receptor c-met. Figure 6D demonstrates that inactivation of HGF/SF significantly reduced the invasion of VB6, CA1 and 5PT cells through Matrigel (*P*=0.0014, 0.0159, 0.0012, respectively). Following HGF/SF inhibition, the level of invasion was similar to that produced by FCM, suggesting that the invasion-promoting effect of MCM was mediated by HGF/SF.

These data are the first to show that invasion of SCC may be due partly to a double paracrine effect, resulting in proinvasive release of HGF/SF from stromal myofibroblasts.

## DISCUSSION

Accumulation of fibroblast-like cells, including myofibroblasts, is frequently observed associated with the edge of an actively expanding tumour mass (Martin *et al*, 1996; Emura *et al*, 2000). Such a phenomenon has been demonstrated, to different extents, in a variety of tumours and there is increasing evidence that tumour stroma may promote tumour progression (Liotta and Kohn, 2001; Pupa *et al*, 2002). Interactions between epithelial cells and fibroblasts have a major role in many biological processes and it follows that the interactions between tumour cells and neighbouring myofibroblasts may be biologically significant, probably mediated by soluble factors such as growth factors and cytokines. This has been demonstrated previously in breast cancer where TGF-*β*1 produced by breast cancer cells activates normal breast stromal fibroblasts and promotes them to produce proteases (Ronnov-Jessen and Petersen, 1993; Sieuwerts *et al*, 1998). Similar interactions have been shown in prostatic carcinomas (Olumi *et al*, 1999; Webber *et al*, 1999), and in the fibrosis observed in organs such as the kidney (Lewis and Norman, 1998) and liver (Kinnman and Housset, 2002).

In the present study, we examine potential interactions between squamous carcinoma cells and primary fibroblasts. We show that stromal cells in SCC *in vivo* often express SMA, indicating a myofibroblastic phenotype (Table 1; Figure 1). Such cells are most commonly found at the invasive margin, directly abutting tumour cells but are absent in areas distant from tumour. Furthermore, myofibroblasts were not detected in benign mucosal polyps. These data are consistent with the possibility of a tumour-derived, diffusible factor that promotes fibroblast-to-myofibroblast transdifferentiation.

A number of cytokines including PDGF, IL-4, insulin-like growth factor II and TGF-*β*1 may be involved in the transdifferentiation of fibroblasts to myofibroblasts, and these can be derived from several cell types. In addition, mast cell-derived histamine and tryptase has been reported to induce SMA expresson in fibroblasts (Gailit *et al*, 2001). However, it is generally accepted that TGF-*β*1 has a key role in inducing myofibroblast differentiation, and high levels of the cytokine are usually associated with MF-containing lesions (Tuan and Nichter, 1998). TGF-*β*1 is frequently detectable in SCC, particularly in the more advanced stages of tumour progression, and relatively high concentrations of TGF-*β*1 are usually found in tumour stroma (Pasche, 2001). Recently, Bauer *et al* (2002) showed that keratinocytes genetically modified to produce activated TGF-*β*1 induced collagen type I gene expression in dermal fibroblasts in a coculture system. The role of TGF-*β*1 in SCC is complex and studies suggest that TGF-*β*1 has biphasic actions on tumour cells, having an important negative growth effect in the early stages of carcinogenesis, but at later stages enhancing invasion and metastasis through epigenetic mechanisms (Akhurst and Balmain, 1999; Akhurst and Derynck, 2001). However, most studies have concentrated on the direct effect of TGF-*β*1 on tumour cells. Our data suggest that a possible indirect tumour-promoting effect of SCC-derived TGF-*β*1 may be in generating a myofibroblastic stroma, which in turn modulates invasion in a paracrine manner.

Myofibroblasts may promote tumour progression in a number of different ways. They upregulate the expression of serine and matrix metalloproteinases, which degrade and remodel extracellular matrix, possibly potentiating cell invasion and migration (Sieuwerts *et al*, 1998). In addition, Ramos *et al* (1997) showed that peritumour FCM upregulated the expression of the integrin *α*v*β*6 in SCC cells, and we have previously demonstrated that *de novo* expression of this integrin promotes invasion of oral carcinoma (Thomas *et al*, 2001a, 2001b). Myofibroblasts also secrete interstitial matrix, as well as numerous soluble mediators of inflammation and growth factors, including HGF/SF (Powell *et al*, 1999a, 1999b). The latter cytokine was originally identified as a potent mitogen for hepatocytes, but was also identified independently as a scatter factor (SF), a secretory protein of fibroblasts and smooth muscle cells that dissociates and induces motility of epithelial cells. Scatter factor and HGF were later found to be identical, hence the current name HGF/SF. The cytokine modulates its effects through the c-met tyrosine kinase receptor and misregulated expression of both cytokine and receptor is a common finding in many tumour types (Trusolino and Comoglio, 2002). Although c-met is generally expressed in oral SCC *in vivo* and *in vitro* (Bennett *et al*, 2000; Morello *et al*, 2001), it is not commonly mutated to a constitutively active form, and is not tumour-promoting *per se* in the absence of ligand. Hepatocyte growth factor/scatter factor may induce invasive growth by affecting the activity and expression of cadherins, integrins and matrix metalloproteinases. This results in disruption of intercellular junctions, dissolution of epithelial basement membrane and altered integrin interactions with extracellular matrix (Trusolino and Comoglio, 2002). Fibroblast-derived HGF/SF has been shown to stimulate invasion and migration in a number of tumour types including squamous cell carcinoma (Matsumoto *et al*, 1994; Uchida *et al*, 2001), and we have demonstrated previously that exogenous HGF/SF induces expression of the type IV collagenases MMP-2 and -9 in squamous carcinoma cells (Bennett *et al*, 2000). The latter observation suggests a possible mechanism for the HGF/SF-dependent invasion through basement membrane-like Matrigel (which comprises predominantly type IV collagen) described in this study. In addition, we have shown more recently that HGF/SF regulates integrin function in oral SCC cells (Poomsawat *et al*, 2003).

Several other paracrine interactions between keratinocytes and fibroblasts have been demonstrated previously. For example, it has been suggested that PDGF-activated stromal cells maintain elevated keratinocyte proliferation via a paracrine mechanism (Skobe and Fusenig, 1998), and Maas-Szabowski *et al* (2001) showed that IL-1 produced by epidermal keratinocytes induced the expression of KGF by dermal fibroblasts, which in turn stimulated keratinocyte proliferation. Paracrine interactions have also been demonstrated between squamous carcinoma cells and other cell types. Liss *et al* (2001) found tumour-derived TGF-*β*1 and monocyte chemotactic protein-1 attracted and activated monocytes. They suggested that macrophages secreted TNF-alpha and IL-1, which in turn stimulated tumour cells to produce IL-8 and VEGF, the latter cytokine-inducing angiogenesis.

In conclusion, this study shows for the first time that a double paracrine interaction between SCC cells and fibroblasts can exist that results in enhanced tumour invasion. We show that SCC-derived TGF-*β*1 induces a myofibroblastic phenotype and that such cells secrete significantly higher levels of HGF/SF compared with primary fibroblast controls. In turn, HGF/SF promotes invasion of SCC cells through basement membrane proteins. We also confirm that the myofibroblast population is usually located adjacent to the invasive front of SCC. These clinical observations are consistent with the suggestion that the paracrine interactions observed *in vitro* between SCC and fibroblasts may also occur in *vivo*, and emphasises the importance of the stromal contribution to tumour development.

## References

[bib1] Akhurst RJ, Balmain A (1999) Genetic events and the role of TGF beta in epithelial tumour progression. J Pathol 187: 82–90 10341708 10.1002/(SICI)1096-9896(199901)187:1<82::AID-PATH248>3.0.CO;2-8

[bib2] Akhurst RJ, Derynck R (2001) TGF-beta signaling in cancer–a double-edged sword. Trends Cell Biol 11: S44–S51 11684442 10.1016/s0962-8924(01)02130-4

[bib4] Bauer BS, Tredget EE, Marcoux Y, Scott PG, Ghahary (2002) Latent and active transforming growth factor beta1 released from genetically modified keratinocytes modulates extracellular matrix expression by dermal fibroblasts in a coculture system. J Invest Dermatol 119: 456–463 12190870 10.1046/j.1523-1747.2002.01837.x

[bib5] Bennett JH, Morgan MJ, Whawell SA, Atkin P, Roblin P, Furness J, Speight PM (2000) Metalloproteinase expression in normal and malignant oral keratinocytes: stimulation of MMP-2 and -9 by scatter factor. Eur J Oral Sci 108: 281–291 10946762 10.1034/j.1600-0722.2000.108004281.x

[bib6] Bradbury J (1998) A two-pronged approach to the clinical use of HGF. Lancet 351: 272 9471475 10.1016/S0140-6736(05)78259-3

[bib7] Emura M, Ochiai A, Horino M (2000) Development of myofibroblasts from human bone marrow mesenchymal stem cells cocultured with human colon carcinoma cells and TGF beta 1. *In vitro* Cell Dev Biol Anim 36: 77–80 10718362 10.1290/1071-2690(2000)036<0077:domfhb>2.0.co;2

[bib8] Gailit J, Marchese MJ, Kew RR, Gruber BL (2001) The differentiation and function of myofibroblasts is regulated by mast cell mediators. J Invest Dermatol 117: 1113–1119 11710921 10.1046/j.1523-1747.2001.15211.x

[bib9] Goke M, Kanai M, Podolsky DK (1998) Intestinal fibroblasts regulate intestinal epithelial cell proliferation via hepatocyte growth factor. Am J Physiol 274: G809–G818 9612260 10.1152/ajpgi.1998.274.5.G809

[bib10] Kinnman N, Housset C (2002) Peribiliary myofibroblasts in biliary type liver fibrosis. Front Biosci 7: D496–D503 11815289 10.2741/A790

[bib11] Lee H, Douglas-Jones AG, Morgan JM, Jasani B (2002) The effect of fixation and processing on the sensitivity of oestrogen receptor assay by immunohistochemistry in breast carcinoma. J Clin Pathol 55: 236–238 11896082 10.1136/jcp.55.3.236 PMC1769609

[bib12] Lewis MP, Norman JT (1998) Differential response of activated vs. non-activated renal fibroblasts to tubular epithelial cells: a model of initiation and progression? Exp Nephrol 6: 132–143 9567219 10.1159/000020514

[bib13] Liotta LA, Kohn EC (2001) The microenvironment of the tumour–host interface. Nature 411: 375–379 11357145 10.1038/35077241

[bib14] Liss C, Fekete MJ, Hasina R, Lam CD, Lingen MW (2001) Paracrine angiogenic loop between head-and-neck squamous-cell carcinomas and macrophages. Int J Cancer 93: 781–785 11519037 10.1002/ijc.1407

[bib15] Maas-Szabowski N, Stark HJ, Fusenig ME (2001) Keratinocyte growth regulation in defined organotypic cultures through IL-1-induced keratinocyte growth factor expression in resting fibroblasts. J Invest Dermatol 114: 1075–1084 10.1046/j.1523-1747.2000.00987.x 10844548

[bib16] Martin M, Pujuguet P, Martin F (1996) Role of stromal myofibroblasts infiltrating colon cancer in tumor invasion. Pathol Res Pract 192: 712–717 8880872 10.1016/S0344-0338(96)80093-8

[bib17] Matsumoto K, Matsumoto K, Nakamura T, Kramer RH (1994) Hepatocyte growth factor/scatter factor induces tyrosine phosphorylation of focal adhesion kinase (p125FAK) and promotes migration and invasion by oral squamous cell carcinoma cells. J Biol Chem 269: 31807–31813 7527397

[bib18] Morello S, Olivero M, Aimetti M, Bernardi M, Berrone S, Di Renzo MF, Giordano S (2001) MET receptor is overexpressed but not mutated in oral squamous cell carcinomas. J Cell Physiol 189: 285–290 11748586 10.1002/jcp.10010

[bib43] Nishimura K, Kitamura M, Miura H, Nonomura N, Takada S, Takahara S, Matsumoto K, Nakamura T, Matsumiya K (1999) Prostate stromal cell-derived hepatocyte growth factor induces invasion of prostate cancer cell line DU145 through tumor-stromal interaction. Prostate 41: 145–153 10517872 10.1002/(sici)1097-0045(19991101)41:3<145::aid-pros1>3.0.co;2-r

[bib19] Olumi AF, Grossfeld GD, Hayward SW, Carroll PR, Tlsty TD, Cunha GR (1999) Carcinoma-associated fibroblasts direct tumor progression of initiated human prostatic epithelium. Cancer Res 59: 5002–5011 10519415 10.1186/bcr138 PMC3300837

[bib20] Pasche B (2001) Role of transforming growth factor beta in cancer. J Cell Physiol 186: 153–168 11169452 10.1002/1097-4652(200002)186:2<153::AID-JCP1016>3.0.CO;2-J

[bib21] Poomsawat S, Whawell SA, Morgan MJ, Thomas GJ, Speight PM (2003) Scatter factor regulation of integrin expression and function on oral epithelial cells. Arch Derm Res 295: 63–70 12750924 10.1007/s00403-003-0388-5

[bib22] Powell DW, Mifflin RC, Valentich JD, Crowe SE, Saada JI, West AB (1999a) Myofibroblasts. I. Paracrine cells important in health and disease. Am J Physiol 277: C1–C19 10409103 10.1152/ajpcell.1999.277.1.C1

[bib23] Powell DW, Mifflin RC, Valentich JD, Crowe SE, Saada JI, West AB (1999b) Myofibroblasts. II. Intestinal subepithelial myofibroblasts. Am J Physiol 277: C183–C201 10444394 10.1152/ajpcell.1999.277.2.C183

[bib24] Pupa SM, Menard S, Forti S, Tagliabue E (2002) New insights into the role of extracellular matrix during tumour onset and progression. J Cell Physiol 192: 259–267 12124771 10.1002/jcp.10142

[bib26] Ramos DM, Chen BL, Boylen K, Stern M, Kramer RH, Sheppard D, Nishimura SL, Greenspan D, Zardi L, Pytela R (1997) Stromal fibroblasts influence oral squamous-cell carcinoma cell interactions with tenascin-C. Int J Cancer 72: 369–376 9219848 10.1002/(sici)1097-0215(19970717)72:2<369::aid-ijc28>3.0.co;2-9

[bib27] Ronnov-Jessen L, Petersen OW (1993) Induction of alpha-smooth muscle actin by transforming growth factor-beta 1 in quiescent human breast gland fibroblasts. Implications for myofibroblast generation in breast neoplasia. Lab Invest 68: 696–707 8515656

[bib29] Ronnov-Jessen L, Peterson OW, Bissell MJ (1996) Cellular changes involved in converson of normal to malignant breast:importance of the stromal reaction. Physiol Rev 76: 69–125 8592733 10.1152/physrev.1996.76.1.69

[bib30] Rowley DR (1998) What might a stromal response mean to prostate cancer progression? Cancer Metast Rev 17: 411–419 10.1023/a:1006129420005 10453285

[bib31] Serini G, Gabbiani G (1999) Mechanisms of myofibroblast activity and phenotypic modulation. Exp Cell Res 250: 273–283 10413583 10.1006/excr.1999.4543

[bib32] Sieuwerts AM, Klijn JG, Henzen-Logmand SC, Bouwman I, Van Roozendaal KE, Peters HA, Setyono-Han B, Foekens JA (1998) Urokinase-type-plasminogen-activator (uPA) production by human breast (myo) fibroblasts *in vitro*: influence of transforming growth factor-beta(1) (TGF beta(1)) compared with factor(s) released by human epithelial-carcinoma cells. Int J Cancer 76: 829–835 9626349 10.1002/(sici)1097-0215(19980610)76:6<829::aid-ijc11>3.0.co;2-y

[bib33] Skobe M, Fusenig NE (1998) Tumorigenic conversion of immortal human keratinocytes through stromal cell activation. Proc Natl Acad Sci USA 95: 1050–1055 9448283 10.1073/pnas.95.3.1050 PMC18668

[bib42] Sugiyama M, Speight PM, Prime SS, Watt FM (1993) Comparison of integrin expression and terminal differentiation capacity in cell lines derived from oral squamous cell carcinoma. Carcinogenesis 14: 2171–2176 7693359 10.1093/carcin/14.10.2171

[bib34] Thomas GJ, Lewis MP, Hart IR, Marshall JF, Speight PM (2001a) α V β 6 integrin promotes invasion of squamous carcinoma cells through up-regulation of matrix metalloproteinase-9. Int J Cancer 92: 641–650 11340566 10.1002/1097-0215(20010601)92:5<641::aid-ijc1243>3.0.co;2-p

[bib35] Thomas GJ, Lewis MP, Whawell SA, Russel A, Hart IR, Speight PM, Marshall JF (2001b) *α*v*β*6 integrin promotes invasion and migration in squamous carcinoma cells. J Invest Dermatol 117: 67–73 11442751 10.1046/j.0022-202x.2001.01379.x

[bib36] Tlsty TD, Hein PW (2001) Know thy neighbour: stromal cells can contribute oncogenic signals. Curr Opin Genet Dev 11: 54–59 11163151 10.1016/s0959-437x(00)00156-8

[bib37] Trusolino L, Comoglio PM (2002) Scatter-factor and semaphoring receptors: cell signaling for invasive growth. Nat Rev Cancer 2: 289–300 12001990 10.1038/nrc779

[bib38] Tuan TL, Nichter LS (1998) The molecular basis of keloid and hypertrophic scar formation. Mol Med Today 4: 19–24 9494966 10.1016/S1357-4310(97)80541-2

[bib25] Tuxhorn JA, Ayala GE, Rowley DR (2001) Reactive stroma in prostate cancer progression. J Urology 166: 2472–2483 11696814

[bib39] Uchida D, Kawamata H, Omotehara F, Nakashiro Ki, Kimura-Yanagawa T, Hino S, Begum MM, Hoque MO, Yoshida H, Sato M, Fujimori T (2001) Role of HGF/c-met system in invasion and metastasis of oral squamous cell carcinoma cells *in vitro* and its clinical significance. Int J Cancer 93: 489–496 11477552 10.1002/ijc.1368

[bib40] Wakefield LM, Roberts AB (2002) TGF-beta signaling: positive and negative effects on tumorigenesis. Curr Opin Genet Dev 12: 22–29 11790550 10.1016/s0959-437x(01)00259-3

[bib41] Webber MM, Trakul N, Thraves PS, Bello-DeOcampo D, Chu WW, Storto PD, Huard TK, Rhim JS, Williams DE (1999) A human prostatic stromal myofibroblast cell line WPMY-1: a model for stromal–epithelial interactions in prostatic neoplasia. Carcinogenesis 20: 1185–1192 10383888 10.1093/carcin/20.7.1185

